# The Highlighted Roles of Metabolic and Cellular Response to Stress Pathways Engaged in Circulating hsa-miR-494-3p and hsa-miR-661 in Alzheimer’s Disease

**DOI:** 10.29252/ibj.25.1.62

**Published:** 2020-08-31

**Authors:** Zohreh Hojati, Farzaneh Omidi, Moein Dehbashi, Bahram Mohammad Soltani

**Affiliations:** 1 Division of Genetics, Department of Cell and Molecular Biology and Microbiology, Faculty of Biological Science and Technology, University of Isfahan, Isfahan 8174673441, Iran;; 2 Department of Genetics, Faculty of Biological Sciences, Tarbiat Modares University, Tehran, Iran

**Keywords:** Alzheimer’s disease, Serum, Circulating microRNAs

## Abstract

**Background:**

Among different roles of miRNAs in AD pathogenesis, hsa-miR-494-3p and hsa-miR-661 functions are poorly understood.

**Methods:**

To obtain the gene targets, gene networks, gene ontology, and enrichment analysis of the two miRNAs, some web servers were utilized. Furthermore, the expressions of these miRNAs were analyzed by qRT-PCR in 36 blood sera, including 18 Alzheimer’s patients and 18 healthy individuals.

**Results:**

The *in silico* analysis demonstrated the highlighted roles of metabolic and cellular response to stress pathways engaged in circulating hsa-miR-494-3p and hsa-miR-661 in AD. The qRT-PCR analysis showed that the downregulated expression level of hsa-miR-661 was statistically significant (*p* < 0.05). Also, the ROC curve of hsa-miR-661 displayed the significant AUC (*p* = 0.01).

**Conclusion:**

Based on our findings, the metabolic and cellular responses to stress pathways are closely connected to these two miRNAs functions. Besides, the qRT-PCR and Roc curve determined hsa-miR-661 could be as a biomarker for diagnosis or prognosis of AD patients.

## INTRODUCTION

One of the oncoming neurodegenerative disorders affecting elderly people is AD^[^^1^^]^. The impediment in reminding short-term incidents, speaking, and reasoning abilities, as well as some symptoms, including disorientation, behavioral issues, and changes in personality are the main characteristics of AD. Based on statistics, over 46.8 million people suffer from AD across the world, and this number is estimated to rise to 131.5 million by 2050^[^^2^^]^. In this illness, two important pathologic signs are observed in the brain of affected patients. The first sign encompasses the formation of extracellular amyloid plaques and the second is the growing hyperphosphorylated Tau proteins, as the intracellular neurofibrillary tangles. Nowadays, the noninvasive diagnostic tests, which are able to detect nucleic acids, particularly miRNAs, are in spotlight^[^^3^^]^. These non-coding RNAs (~22-25 nucleotides in length) are involved in post-transcriptional regulation of gene expression in the cells. They are classified as (i) tissue and cellular niche-specific and (ii) circulating small non-coding RNAs for cell-to-cell communications^[^^4^^]^. Notably, miRNAs have been detected to be deregulated in the blood, plasma, serum, CSF, extracellular fluid, and brain tissues of neurodegenerative patients such as AD^[^^5^^]^. 

Among circulating miRNAs, the functions of hsa-miR-494-3p and hsa-miR-661 are poorly understood in AD. Both are expressed in cortex, cerebellum, tibial nerve and detected in whole blood and serum (https://www.genecards.org/). Hsa-miR-494-3p affects cell proliferation, and adhesion as well as cell invasion in glioblastoma multiforme tumors. The presence of this miRNA in glioblastoma multiforme biopsies has been confirmed^[^^6^^]^. Hsa-miR-494-3p induces a cellular senescence caused by the downregulation of BMI1^[^^7^^]^. Some reports have demonstrated that hsa-miR-494-3p is involved in human autism^[^^8^^]^, therapy-resistant epilepsy patients, epileptic rats and serum biomarker^[^^9^^,^^10^^]^. The hsa-miR-661 is, however, involved in blood mononuclear cells, Alzheimer’s patients cortex^[^^11^^]^, hepatocellular carcinoma^[^^12^^]^, type 2 diabetes^[^^13^^]^ and breast cancer cell invasion^[^^14^^]^. In the present study, we selected two lesser known miRNAs in AD pathogenesis with few clues in brain and central nervous diseases. Thus, we tried to predict more potential targets of hsa-miR-494-3p and hsa-miR-661 to gain a better understanding of the underlying targeted genes, enrichment analysis, and signaling pathways. We also attempted to study the expression levels of the two aforesaid miRNAs in the serum samples of AD patients and healthy controls using qRT-PCR.

## MATERIALS AND METHODS

**Network and enrichment analysis**

The publicly available databases, namely TargetScan (http://www.targetscan.org/vert_71/), miRTargetLink Human (https://ccb-web.cs.uni-saarland.de/ mirtargetlink/), and mirDIP (http://ophid.utoronto.ca/ mirDIP/index.jsp) were utilized. The targets of hsa-miR-494-3p and miR-661 were achieved based on strong evidence, weaker evidence, and predicted interactions from miRTargetLink. Also, the targets of the two miRNAs were obtained according to the score class (very high, high, and medium) from mirDIP. Additionally, STRING 10.5 (https://string-db.org/), Kyoto Encyclopedia of Genes and Genomes (KEGG) Biological Pathway (http://www.genome.jp/), and ShinyGO v0.61 (Gene Ontology Enrichment Analysis + more; http://bioinformatics.sdstate.edu/go) were used to identify the gene networks and gene ontology enrichment analysis, by *p* value cutoff = 0.05 for false discovery rate.

**Serum samples**

Participants included in this study were Alzheimer’s patients residing at the Sadeghyeh Welfare Organization (Isfahan, Iran) between December 2016 and February 2017. A total of 36 blood samples, including 18 AD patients and 18 healthy individuals, were collected; their sera were separated and kept at -80 °C until use. NINDS-ADRDA and the revised criteria (pertained to the National Institute on Aging-Alzheimer’s Association) were used for the diagnosis of AD patients^[15,16]^. The participated patients had not previously been prescribed with any treatment of the disease. 

**RNA isolation**

All the RNAs (including miRNA) were isolated by miRCURY™ RNA Isolation Kit-Biofluids (Exiqon, Denmark) from serum samples according to the manufacturer’s instruction. The ratio of A260/A280 was considered as the purity of the RNA. The suitable ratio was 1.8-2.1 for the isolated RNAs. Also, hsa-miR-451^[^^17^^,^^18^^]^ and UniSp6 (recommended by kit) were used as the most endogenous internal control and the spike-in control, respectively.

**cDNA synthesis, qRT-PCR, and PAGE electrophoresis**

cDNAs synthesis for hsa-miR-494-3p, hsa-miR-661, and hsa-miR-451 (internal control) were performed by miRCURY LNA™ Universal RT microRNA PCR (Exiqon), as stated by the manufacturer. UniSp6, the RNA spike-in template, was used as a positive control. The cDNA products were incorporated into a master mix composed of 10 pmol/μl of hsa-miR-494-3p, hsa-miR-661, and hsa-miR-451 DNA primers (Exiqon) and 2 U of ExiLEN SYBR® Green master mix (Exiqon). RT reaction (20 µl) was diluted 20×, and 4 µl of the diluted cDNA was used in 10 µl of PCR amplification reactions. A non-template control was added to verify the specificity of the qRT-PCR. Reactions of qRT-PCR were carried out using Opticon Monitor 3 (Bio-Rad Laboratories Inc., Hercules, CA, USA), and all the reactions were conducted in triplicate. Data of qRT-PCR were assessed according to the 2^-ΔΔCT^ method. All specific amplicons resulted from qRT-PCR were loaded and electrophoresed on 12% non-denaturing PAGE in 1X TBE buffer along with 50 bp of DNA ladder (Fermentas, USA) and visualized by silver staining. 

**Statistical analysis**

Statistical tests were executed by SPSS (version 21, IBM Corporation, Armonk, NY, USA). Student's independent *t*-test was carried out to compare the quantitative expression level of hsa-miR-494-3p and hsa-miR-661 between different groups of patients. For distinguishing between AD patients and healthy controls, the ROC curve analysis was performed. The AUC was measured to determine the diagnostic accuracy of the identified miRNAs. For all analyses, *p* values < 0.05 were considered statistically significant.

**Ethics statements**

The research was approved by the Ethics Committee of Tarbiat Modares University (Tehran), University of Tehran, and University of Isfahan (Isfahan, Iran). The ethical code number dedicated to the study was 98/50297. Informed consent was received from all the participants before beginning the study. In case of AD patients, their legal guardians filled and signed the consent form.

## RESULTS

***In silico*** **results**

Based on the predicted targets of hsa-miR-494-3p in mirDIP server, it was notable that integrated scores ranged between 0.86 and 0.014. Also, in this server, the predicted targets of hsa-miR-661 were in the range of 0.68-0.014, by the integrated scores. In TargetScan 7.1, the predicted targets of hsa-miR-494-3p and hsa-miR-661 showed the total context++ score between -1.39 and -0.02 and between -2.25 and -0.02, respectively. According to the KEGG server and the GO Biological process option, all the 12751 and 76471 predicted target genes, respectively were pertained to hsa-miR-494-3p and hsa-miR-661 and located mainly on the chromosomes 1, 19, 2, and 11 by *p* = 1.9e-113 and *p* = 5.5e-144, respectively. In addition, metabolic pathways and the cellular response to stress pathway for hsa-miR-494-3p and hsa-miR-661 were engaged as the top predicted pathways by *p* = 8.8e-42 and *p *= 5.2e-38 and *p* = 1.6e-156 and *p* = 1.8e-165, respectively.

**Expression analysis of hsa-miR-494-3p and hsa-miR-661 in AD serum samples**

**Fig. 1:**
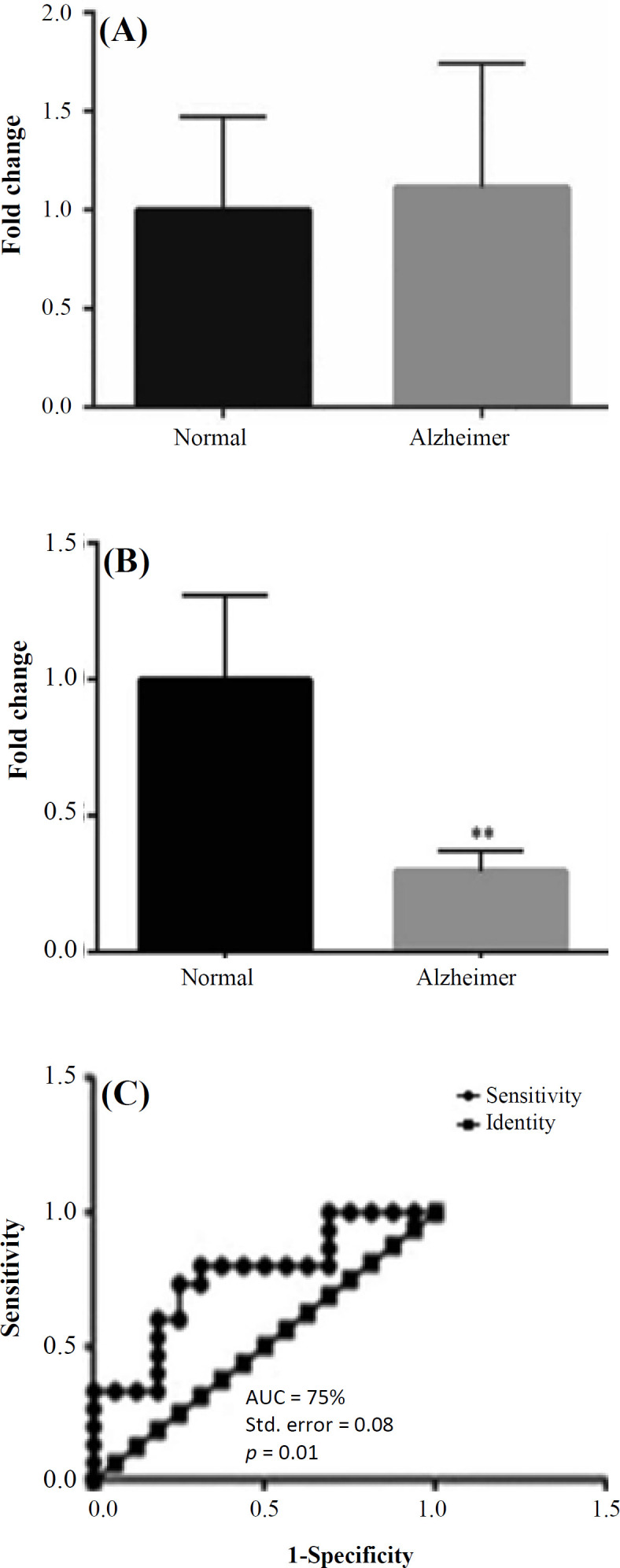
The fold change graphs of hsa-miR-494-3p and hsa-miR-661. (A) The hsa-miR-494-3p was upregulated in AD patients as 0.34 fold change compared with the healthy controls (*p* = 0.86); (B) the hsa-miR-661 was downregulated in AD patients as three fold change compared with the healthy controls (*p* = 0.01); (C) ROC curves were drawn and AUC was calculated to evaluate and compare the diagnostic and prognostic potential of serum hsa-miR-661 in AD patients versus healthy controls. The calculations showed AUC = 75%, standard error = 0.08, and *p* value = 0.01; thus, hsa-miR-661 possessed the potential to be as a biomarker

Based on the real-time PCR conclusions, the amplification curve of hsa-miR-451 with average 20.62 Ct and 84 °C melting curve by single pick were observed. The amplification curve of hsa-miR-494-3p and hsa-miR-661 had 35 and 38 Ct averages of AD samples in comparison with 37 and 35 Ct averages of healthy controls, respectively. Also, melting curves of 83 °C and 84 °C were observed for hsa-miR-494-3p and hsa-miR-661 by single picks, respectively. The electrophoretic separation of products on 12% non-denaturing PAGE showed one specific amplified product for three miRNAs, including the hsa-miR-494-3p, hsa-miR-661, and internal control of hsa-miR-451. Among the studied AD patients, the expression level of hsa-miR-494-3p and hsa-miR-661 showed upregulation and downregulation, respectively, in comparison with the healthy controls (Fig. 1a and 1b). Also, statistical analyses revealed that the upregulated expression of hsa-miR-494-3p was not significant (*p* = 0.86), but the downregulated expression of hsa-miR-661 was significant (*p* = 0.01). 

**ROC curve for introducing the possible biomarkers**

In order to evaluate the potential biomarkers of hsa-miR-494-3p and hsa-miR-661 among AD patients and healthy controls and to investigate the specificity and sensitivity of these miRNAs, ROC curve was analyzed. The analysis demonstrated that hsa-miR-661 had the potential for introducing as a biomarker (*p* = 0.01; Fig. 1c). In details, the AUC and standard error for hsa-miR-661 were 75% and 0.08, respectively. The ROC curve showed that hsa-miR-494-3p had a lower AUC than hsa-miR-661. 

## DISCUSSION

Delayed diagnosis of an illness is a major obstacle to find the successful treatment^[^^19^^]^. In this sense, the development of new molecular biomarkers is essential for prognostic and diagnostic purposes. Biomolecular studies have shown that miRNAs possess the great potential of prognosis and diagnosis^[^^20^^]^, and they can illuminate the path of treatment in many diseases. 

Several studies around the subject of miRNAs profiling have been carried out, and the alteration in expression levels of AD-associated miRNAs have been analyzed^[^^21^^,^^11^^]^. The database and experimental results pertained to circulating and expressing hsa-miR-494-3p and hsa-miR-661 in some diseases persuaded us to investigate the expression level of the aforesaid miRNAs in the sera of AD patients. Using our network and enrichment analysis of these two miRNAs, two pathways, including metabolic and cellular response to stress pathways, were highlighted in the AD pathogenesis. In accordance with these results, BACE1 is an enzyme involved in initiating b-amyloid generation^[^^22^^]^. Other reports have suggested that BACE1 is a stress response protein with elevated levels in oxidative stress^[^^23^^]^, hypoxia^[^^24^^]^, ischemia^[^^25^^]^, apoptosis^[^^26^^]^, and traumatic brain injury^[^^27^^]^. Our bioinformatics analysis suggested one of the genes predicted as a target of miR-661 is BACE1. This result seems to prove the involvement of miR-661 in cellular response to the stress pathway. ATF6 is a 90-kDa type II transmembrane protein with the N-terminal domain.

located in the cytoplasm. This protein is processed at or close to the cytosolic face of the ER membrane in response to stress^[^^28^^]^. ATF6 has been reported to be proteolyzed by S1P and S2P, which are known to be processing enzymes as sterol regulatory element-binding proteins^[^^29^^]^. Researchers have indicated that S1P and S2P are required for the ER stress response as well as for the lipid synthesis (as a metabolic pathway), and also that cleavage of ATF6 at the ER membrane is highly controlled by these proteases during ER stress^[^^30^^]^. Enrichment analysis and target prediction have demonstrated that S1PR1 and S1PR2 are predicted targets of hsa-miR-494-3p and hsa-miR-661 in metabolic pathways and cellular response to stress pathway, respectively. O’Connor *et al*.^[^^31^^]^ and Mouton-Liger *et al*.^[^^31^^]^ have revealed that eIF2-alpha, as one of the ER stress pathway component, is phosphorylated and upregulated in the frontal and temporal cortex in human AD patients, respectively. The eIF2-alpha was obtained as targets of hsa-miR-494-3p and hsa-miR-661 in our *in silico* analysis. 

**Fig. 2:**
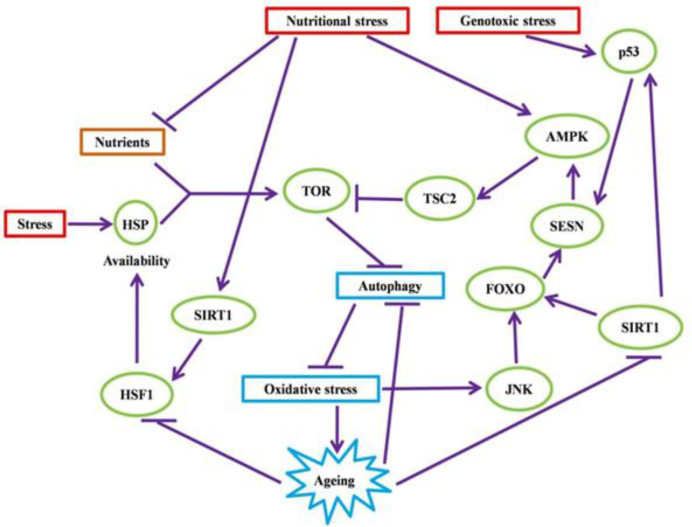
The crossroad of metabolism, protein homeostasis, and cellular response to the stress pathway and ageing

It has been investigated that the low concentrations of ROS have ability to induce the expression of antioxidant enzymes and other defense mechanisms. AD, PD, HD, ALS, and FRDA all are categorized as “protein conformational diseases”, influencing some elderly people throughout the world. Unfolded proteins are responded by chaperons to salvage misfolded proteins, break up aggregates, and assist in their folding process. Those proteins that cannot be salvaged by refolding are given to the proteasome and recycled. Under dysfunctional aggregation of proteins, multiple metabolic derangements often happen in connection with the excessive production of ROS and oxidative stress^[^^32^^]^ (Fig. 2). 

From experimental viewpoint, we can confirm the previous results pertained to circulating hsa-miR-494-3p. The qRT-PCR analysis of hsa-miR-494-3p in the present study showed the upregulation of this miRNA in the serum of AD patients in comparison with healthy controls, but it was not statistically significant. Concerning hsa-miR-661, the qRT-PCR results demonstrated that this miRNA was significantly downregulated in the serum of AD patients in comparison of healthy individuals as a circulating miRNA. Roc curve showed that this miRNA possesses the potential to be a biomarker for diagnosis or prognosis purposes in AD patients. 

Our *in silico* analyses reveal that the metabolic and cellular response to stress pathways are closely connected and have crossroad that affect directly or indirectly the pathogenesis of AD. However, further experimental verifications are highly recommended to examine our results.
